# hERG1 Potassium Channel Expression in Colorectal Adenomas: Comparison with Other Preneoplastic Lesions of the Gastrointestinal Tract

**DOI:** 10.3390/cimb44030089

**Published:** 2022-03-17

**Authors:** Elena Lastraioli, Jessica Iorio, Federica Petrelli, Anna Tomezzoli, Serena Battista, Maria Raffaella Ambrosio, Mariella Chiudinelli, Federica De Salvatore, Luca Messerini, Vincenzo Villanacci, Luca Saragoni, Annarosa Arcangeli

**Affiliations:** 1 Department of Experimental and Clinical Medicine, University of Florence, Viale GB Morgagni 50, 50134 Florence, Italy; jessica.iorio@unifi.it (J.I.); luca.messerini@unifi.it (L.M.); annarosa.arcangeli@unifi.it (A.A.); 2 Pathology Division, Azienda Ospedaliero-Universitaria Senese, Viale M Bracci 16, 53100 Siena, Italy; federica.petrelli@uslnordovest.toscana.it (F.P.); maradot@libero.it (M.R.A.); 3 Pathology Division, Borgo Trento Hospital, Piazzale A Stefani 1, 37134 Verona, Italy; annatomez@hotmail.com; 4 Pathology Department, “S. Maria della Misericordia Hospital”, Friuli-Venezia Giulia, 33100 Udine, Italy; serena.battista@asufc.sanita.fvg.it; 5 Pathology Division, Esine Hospital, ASST della Valcamonica, Via A Manzoni 142, 25040 Esine, Italy; mariel.chiudinelli@gmail.com; 6 Institute of Pathology, ASST Spedali Civili di Brescia, Piazzale Spedali Civili 1, 25123 Brescia, Italy; federica.desalvatore@libero.it (F.D.S.); villanac@alice.it (V.V.); 7 Pathology Division, Morgagni-Pierantoni Hospital, Via C Forlanini 34, 47121 Forlì, Italy; l.saragoni@ausl.fo.it

**Keywords:** hERG1 potassium channels, colorectal adenomas, gastric dysplasias, Barrett’ s esophagus

## Abstract

Preneoplastic lesions represent a useful target for early diagnosis and follow-up of gastrointestinal malignancies. hERG1 channel expression was tested by immunohistochemistry (IHC) in a cohort of colorectal adenoma samples belonging to Italian subjects. Overall, hERG1 was expressed in 56.5% of cases with both high staining intensity and a high percentage of positive cells. Moreover, hERG1 was expressed in a higher percentage of dysplastic adenomas with respect to hyperplastic lesions, and the proportion of positive samples further increased in patients with high-grade dysplasia. Comparing hERG1 expression in other preneoplastic lesions of the GI tract (gastric dysplasia and Barrett’s esophagus), it emerged that in all the conditions, hERG1 was expressed with a diffused pattern, throughout the cell, with variable staining intensity within the samples. The highest expression was detected in gastric dysplasia samples and the lowest in Barrett’s esophagus at similar levels observed in colorectal adenomas. Our results show that hERG1 is aberrantly expressed in human preneoplastic lesions of the gastrointestinal tract and has a different pattern of expression and role in the different sites. Overall, the detection of hERG1 expression in preneoplastic lesions could represent a novel diagnostic or prognostic marker of progression in the gastrointestinal tract.

## 1. Introduction

The gastrointestinal (GI) tract can be affected by precancerous lesions likely to evolve in cancer [1,2]. GI precancerous lesions can develop throughout the whole upper and lower GI tract. Barrett’s esophagus, chronic gastritis with or without *Helicobacter pylori* infection, atrophic gastritis, intestinal metaplasia of the gastric mucosa, gastric epithelial dysplasia, and gastric polyps might arise in the upper digestive tract [3]. At the same time, colorectal adenomas, inflammatory bowel disease, and hereditary non-polyposis lesions of the colon mainly affect the lower gastrointestinal tract [4].

The European Society of Gastrointestinal Endoscopy (ESGE) and American Gastroenterological Association (AGA) guidelines classify patients with preneoplastic lesions in different risk subgroups based on histology [5,6]. Nevertheless, definitive molecular factors that identify which early gastrointestinal and colorectal lesions are most likely to relapse are still lacking [5,6]. According to Globocan 2020 estimates, GI tract tumors still represent a major health problem since they all belong to the top 10 most lethal cancers: in particular, mortality rates for Colorectal Cancer (CRC) are located in the third position, Gastric Cancer (GC) is the fifth leading cause of mortality from cancer worldwide, and Esophageal Cancer (EC) ranks 8 (source: Globocan 2020, https://gco.iarc.fr accessed on 10 January 2022). For these reasons, searching for novel and powerful biomarkers in GI precancerous lesions is mandatory to identify at-risk lesions.

Several studies carried out in different solid tumors demonstrated that Ion Channels and Transporters (ICT) are frequently misexpressed and play important roles in regulating cancer cell behavior. In this context, after proper validation, ICT could represent novel cancer biomarkers [7,8]. Among ion channels, potassium channels are the most frequently deregulated in human tumors, as witnessed by several reports (reviewed in [7]). In particular, hERG1 is an outward rectifying K^+^ channel belonging to the EAG family encoded by the *ether*-à-*go*-*go* related gene 1 (*KCNH2*). It was shown long time ago that this channel is often overexpressed in neoplastic cell lines. Subsequently, it was demonstrated that hERG1 is also aberrantly expressed in several primary solid tumors [7,9,10,11] and some precancerous lesions [12,13]. In physiological conditions, hERG1 channels mediate the potassium current (I_Kr_) responsible for the rapid repolarizing phase that follows cardiac action potential.

This study aimed to evaluate hERG1 expression in colorectal adenomas (CRA) and to compare it with other GI lesions such as Barrett’s esophagus (BE) and gastric dysplasia (GD).

## 2. Materials and Methods

- Tissue collection

We collected 69 colorectal adenoma paraffin-embedded samples (44 males and 25 females, mean age 67.9 years, range 38–86) from different institutions belonging to GIRCG and participating in the study (Department of Clinical and Experimental Medicine, University of Florence; Pathology Division, Azienda Ospedaliero-Universitaria Senese; Insitute of Pathology, Spedali Civili, Brescia; Pathology Division, Esine Hospital, ASL Vallecamonica Sebino; Pathology Division, Borgo Trento Hospital, Verona; Pathology Division, Morgagni-Pierantoni Hospital, Forlì; Pathology Division, Azienda Sanitaria Universitaria Friuli Centrale, Udine). Paraffin-embedded samples were retrieved after the proper selection was carried out, interrogating the databases of the above-mentioned institutions selecting cases of the last ten years (2012–2021).

Diagnosis and histological grading were assessed using standard criteria by experienced pathologists in each institution (L.M., M.R.A., F.P., F.D.S., V.V., M.C., A.T., L.S. and S.B.). Ki67 was not routinely used, except in the distinction and exact definition of low and high grades of dysplasia.

Moreover, 127 BE and 101 gastric dysplasia samples were also analyzed.

The studies were approved by the local Ethical Committee following current guidelines about retrospective observational studies in biological samples, and for each patient, written informed consent was obtained.

- Immunohistochemistry (IHC)

IHC was performed as previously reported [14] using an anti- hERG1 monoclonal antibody (MCK Therapeutics, Florence, Italy; patent number WO2016020483A1) at 1:200 dilution. Slides were incubated overnight at 4 °C, and immunostaining was performed with a commercially available kit (PicTure Max kit, Invitrogen; Carlsbad, CA, USA).

- IHC slides evaluation

hERG1 expression was estimated as the percentage of positive cells. Samples were analyzed field by field from top left to bottom right, under 40× magnification by two independent investigators (EL and JI) using a Leica DMR light microscope (Leica, Wetzlar Germany) and classified as negative or positive according to the presence of positive cells. A cut-off of 1% was applied to discriminate between negative (≤1%) and positive samples (>1%).

## 3. Results

- hERG1 channels expression in colorectal adenomas

**Figure 1:**
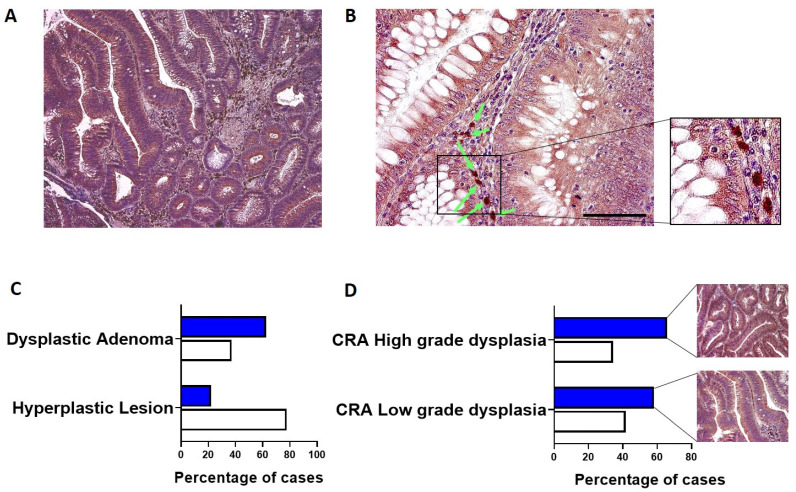
hERG1 potassium channel expression in CRA. (**A**,**B**) representative samples showing a high hERG1 expression in the epithelial cells as well as in plasma cells (see arrows in panel (**B**) and inset to panel (**B**). Scale bar: 100 μm. (**C**) Histogram showing hERG1 expression according to the presence of dysplasia. White bars: hERG1 negative, Blue bars: hERG1 positive. (**D**) Histogram showing hERG1 expression according to the grade of dysplasia. White bars: hERG1 negative, Blue bars: hERG1 positive. Representative slides of the two groups are also shown.

hERG1 expression was analyzed in a group of 69 CRA. Overall, the channel was expressed in an elevated percentage of cases (56.5%, 39 out of 69), and the staining intensity and percentage of positive cells were high. As it can be observed in Figure 1A, hERG1 expression was detected in the cytoplasm and membrane of epithelial cells (highlighted in brown by DAB precipitation) in contrast with nuclei counterstained with hematoxylin (blue-purple). Moreover, at higher magnification (Figure 1B), a certain degree of positivity in the stroma surrounding the adenomatous structures is evident, especially in plasma cells (indicated by arrows).

CRA samples were then divided according to the presence of dysplasia: hERG1 was expressed in a higher percentage of dysplastic adenomas (including both low- and high-grade) when compared to hyperplastic lesions (62.70 vs. 22.20, *p* = 0.035, Fisher Exact Test) (Figure 1C). Additionally, subdividing the samples according to the grade of dysplasia, it emerged that hERG1 was expressed in a greater percentage of high-grade samples with respect to low-grade dysplasia samples (65.7 vs. 58.30).

No association between hERG1 expression and gender or age emerged (*p* = 0.801 and *p* = 1.000, respectively).

- Comparison of hERG1 expression in Barrett’s esophagus, gastric dysplasia, and colorectal adenomas

**Figure 2:**
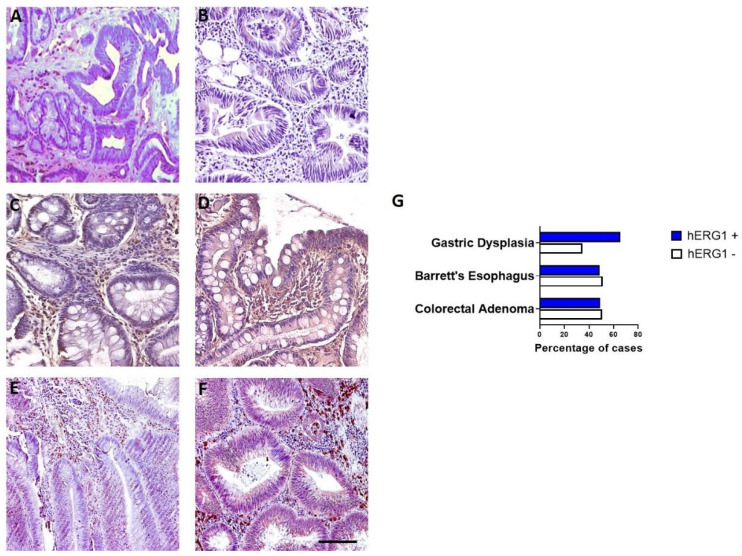
hERG1 potassium channel expression in preneoplastic lesions of the GI tract. (**A**,**B**) Gastric dysplasia. (**C**,**D**) Barrett’s esophagus. (**E**,**F**) colorectal adenoma. Scale bar:100 μm. (**G**) Histogram showing hERG1 expression in the three groups.

The expression of the hERG1 channel has been previously evaluated by our group in gastric dysplasia (GD) [13] and Barrett’ esophagus (BE) [12] samples. Comparing the expression profile, it emerged that in all the conditions, hERG1 was expressed with a diffused pattern, throughout the cell, with variable staining intensity within the samples (Figure 2A–F).

This study confirmed our previous published data regarding hERG1 expression in GD and BE. When comparing the expression in the three preneoplastic conditions, the highest expression was detected in GD samples and the lowest in BE (Figure 2G).

Regarding hERG1 role in GI tumor progression, we already showed that in gastric cancerogenesis, hERG1 channel overexpression occurs at an early stage, being present in intestinal metaplasia frequently existing in Lauren’s intestinal-type lesions [13,15].

## 4. Discussion

In the GI tract, preneoplastic lesions are quite relevant as they can be easily detected and analyzed for their malignant potential. In this context, identifying novel biomarkers could add information and therefore provide novel tools to identify at-risk patients to be treated accordingly. In the last 15 years, it was demonstrated that hERG1 potassium channels represent important determinants of tumor progression in the GI tract and other districts of the body (reviewed in [7]).

This manuscript provides evidence that hERG1 channels are overexpressed in human CRA samples. It is well known that hERG1 is expressed in both CRC cell lines [16] and primary tumors [9,14].

Mounting evidence has highlighted the relevance of potassium channels, particularly hERG1 channels in solid tumors (reviewed in [7]), but little has been reported in preneoplastic lesions.

Within the GI tract, we demonstrated that hERG1 channels are expressed in Barrett’s esophagus [12] and represent useful biomarkers of EC progression. More recently, we demonstrated a similar scenario in GC progression since hERG1 are expressed in GD samples and are associated with the disease progression [13]. Moreover, we also provided evidence that GD patients with high hERG1 expression are characterized by poorer progression-free and overall survival [13].

In this paper, we show that hERG1 channels are expressed in CRA with a similar expression pattern as observed in GD and BE, i.e., diffused to all the cells. hERG1 was expressed in a higher percentage of dysplastic samples, suggesting it might play a major role in the later stages of CRC cancer progression..

hERG1 is not expressed in the healthy mucosa of the three organs [12,15,16] except for oxyntic cells of the gastric mucosa [15]. In preneoplastic lesions, a certain degree of expression is observed. In colorectal adenomas, the expression is comparable to the one observed in metaplastic lesions of the esophagus. In contrast, a higher degree of positivity is observed in the lesions arising in the stomach. Tumors arising in the three sites generally expressed the channel, although to a different extent.

## 5. Conclusions

The data presented here indicate that overall, the detection of hERG1 in preneoplastic lesions of the GI tract could represent a novel tool for early diagnosis and management of the patients. This opportunity aligns with data already published for esophageal and gastric tumors. Nevertheless, it is important to stress that the results reported here represent a pilot study, and confirmation on bigger cohorts of patients is warranted. Moreover, it would be interesting to analyze tumor progression within a single patient, analyzing healthy colon, adenoma, and colorectal cancer, as we previously did for other conditions.

## Data Availability

The data presented in this study are available on request from the corresponding author.
